# MiR-93-5p Promotes Cell Proliferation through Down-Regulating PPARGC1A in Hepatocellular Carcinoma Cells by Bioinformatics Analysis and Experimental Verification

**DOI:** 10.3390/genes9010051

**Published:** 2018-01-22

**Authors:** Xinrui Wang, Zhijun Liao, Zhimin Bai, Yan He, Juan Duan, Leyi Wei

**Affiliations:** 1 State Key Laboratory for Medical Genomics, Shanghai Institute of Hematology, Rui Jin Hospital Affiliated to School of Medicine, Shanghai Jiao Tong University, Shanghai 200025, China; wanxiruqiqi@sina.com; 2 Department of Biochemistry and Molecular Biology, School of Basic Medical Sciences, Fujian Medical University, Fuzhou 350122, China; andy7142003@163.com (Z.B.); hyhyj2002@163.com (Y.H.); duanjuan200609@163.com (J.D.); 3 Department of Clinical Laboratory, Jinjiang Municipal Hospital, Jinjiang 362200, China; 4 School of Computer Science and Technology, Tianjin University, Tianjin 300350, China

**Keywords:** miR-93-5p, PPARGC1A, proliferation, hepatoma, miRNA-mRNA module

## Abstract

Peroxisome proliferator-activated receptor gamma coactivator-1 alpha (PPARGC1A, formerly known as PGC-1a) is a transcriptional coactivator and metabolic regulator. Previous studies are mainly focused on the association between PPARGC1A and hepatoma. However, the regulatory mechanism remains unknown. A microRNA associated with cancer (oncomiR), miR-93-5p, has recently been found to play an essential role in tumorigenesis and progression of various carcinomas, including liver cancer. Therefore, this paper aims to explore the regulatory mechanism underlying these two proteins in hepatoma cells. Firstly, an integrative analysis was performed with miRNA–mRNA modules on microarray and The Cancer Genome Atlas (TCGA) data and obtained the core regulatory network and miR-93-5p/*PPARGC1A* pair. Then, a series of experiments were conducted in hepatoma cells with the results including miR-93-5p upregulated and promoted cell proliferation. Thirdly, the inverse correlation between miR-93-5p and *PPARGC1A* expression was validated. Finally, we inferred that miR-93-5p plays an essential role in inhibiting *PPARGC1A* expression by directly targeting the 3′-untranslated region (UTR) of its mRNA. In conclusion, these results suggested that miR-93-5p overexpression contributes to hepatoma development by inhibiting PPARGC1A. It is anticipated to be a promising therapeutic strategy for patients with liver cancer in the future.

## 1. Introduction

Liver cancer is the third leading cause of cancer death in humans, universally, and hepatocellular carcinoma (HCC) is the most common type of primary liver malignancy [1,2]. The diagnosis, treatment, and overall survival rate of hepatoma have been improved over the past 30 years [3]. However, the lack of diagnostic markers for early detection and limited treatment options available to clinicians increase the risk of lethality and poor prognosis [4,5]. Also, the understanding of its molecular pathogenesis still remains incomplete and fragmentary. Thus, in order to develop an effective therapeutic strategy, it is necessary to conduct a more comprehensive analysis of hepatoma.

It has been clarified that abnormalities in cellular metabolism are closely associated with tumorigenesis and development, including hepatoma [6,7]. Previous studies have reported that mitochondria are key regulators of cellular metabolism, such as aerobic or anaerobic respiration, cellular energy, and fatty acid metabolism [8,9]. Peroxisome proliferator-activated receptor gamma coactivator-1 alpha (PPARGC1A) is a multiple-function transcriptional coactivator that has been identified to be associated with many human diseases, such as type II diabetes mellitus, coronary disease, and other diet-related diseases [10,11]. PPARGC1A is involved in mitochondrial biogenesis and coactivating gene expression by docking to transcription factors (TFs) at the promoters [12]. A recent study has found that the expression of *PPARGC1A* in HCC is significantly lower than in normal liver tissue and hepatocirrhosis. Knockdown of *PPARGC1A* expression in human hepatic cell L-02 cells promotes cancerous tendency [13]. 

MicroRNAs (miRNAs), as a class of short non-coding RNAs [14,15,16], widely participate in the development and progression of human cancers [17,18,19,20]. MiRNAs play as important post-transcriptional regulators by binding to the 3′-untranslated region (3′UTR) of mRNAs and usually suppress target gene expression [21,22]. Accumulating evidence reveal that assessment of differential expression of miRNAs can potentially be used as biomarkers for classification and grading of tumors [23,24,25,26,27,28]. For example, specific miRNAs (miR-21-5p, miR-93-5p, and miR-221-3p) are consistently up-regulated in HCC and play an important role in hepatoma progression [29]. Previous studies have implicated that these miRNAs are closely related with the cellular metabolism process, especially fatty acid metabolism [29,30,31]. Moreover, inhibition of miR-93-5p can significantly suppress HepG2 cell proliferation, migration, and colony formation [32].

In this study, we aim to clarify the role of miR-93-5p and its regulation mechanism on *PPARGC1A* in the hepatoma cells proliferation. Our experimental results demonstrated that the expression of miR-93-5p is inversely correlated with *PPARGC1A* mRNA levels. To be specific, the overexpression of miR-93-5p is capable to promote the proliferation of hepatoma cells. Moreover, we also found that miR-93-5p decreases the expression of TFs CCAAT/Enhancer binding protein beta (CEBPB) by directly binding the 3′UTR of *PPARGC1A*. 

## 2. Methods

### 2.1. Data Mining of Public Resources

GSE57555 is accessible at the National Center for Biotechnology Information (NCBI) Gene Expression Omnibus (GEO) database (http://www.ncbi.nlm.nih.gov/geo/), which provides five HCC patients’ tumor and non-tumor parts tissues microarray datasets. Liver Hepatocellular Carcinoma (LIHC) data in the Cancer Genome Atlas (TCGA public data until 11 June 2015, http://cancergenome.nih.gov/) were also selected for this study (371 HCC tissues and 50 adjacent normal liver tissues, miRNA-seq and RNA-seq level 3 files). Moreover, the differentially expressed genes (DEGs) and microRNAs (DEMs) were identified through fold-change filtering. |Fold Change| ≥ 2 and adjusted *p*-value < 0.01 were used to determine significantly differential expression. In addition, we calculated the Pearson correlation coefficient between gene and miRNA expression levels according to the expression data [33]. The screening criterion was based on correlation coefficient *r* < −0.3 and *p*-value < 0.05. Gene Ontology (GO) analysis were conducted to evaluate the biological functions for this subset of DEGs (*p*-value < 0.01). Simultaneously, we used BisoGenet to visualize the interaction network of DEGs and filtered the hub genes by calculating the value of all the nodes that were carried out by Degree-sorted. For the ChIP-seq analysis, data was available from the GEO database (GSM935623 and GSM935628) and was visualized with the WashU EpiGenome Browser [10,34]. The immunofluorescence (IHC) results of PPARGC1A in HepG2 cells was obtained from The Human Protein Atlas database [35].

### 2.2. Cell Culture and Transfection

Human 293T cells, human hepatic cell L-02, human hepatoma SMMC-7721, Huh-7, SK-Hep-1, HepG2, HCCLM3, and MHCC97H cell lines were purchased from Type Culture Collection of the Chinese Academy of Sciences (Shanghai, China) and cultured in Dulbecco’s modified Eagle’s medium (DMEM; GibcoBRL, Gaithersburg, MD, USA) supplemented with 10% fetal bovine serum (GibcoBRL, Gaithersburg, MD, USA). All cells were maintained at 37 °C in a humidified atmosphere containing 5% CO_2_. MiR-93-5p mimics/inhibitors and the corresponding negative control were purchased from Ribobio (RiboBio Co. Ltd., Guangzhou, China). Cells were transfected at a concentration of 50 nM using Lipofectamine 2000 Reagent (Invitrogen, Carlsbad, CA, USA) according to the manufacturer’s protocol.

### 2.3. Western Blot Analysis

The cells were collected using a plastic cell scraper and lysed in Lysis buffer (20 mM Tris/HCl pH 7.5; 120 mM NaCl; 1 mM EDTA; 1 mM EGTA; 1% Triton X-100; 2.5 mM Sodium pyrophosphate; 1 mM β-Glycerophosphate; 1 mM Na_3_VO_4_) in the presence of protease inhibitors (Sigma-Aldrich, St. Louis, MO, USA). Proteins were dissolved in 5x sample buffer (0.25 M Tris/HCl pH 6.8; 10% (*w*/*v*) SDS; 50% glycerol; 0.5% (*w*/*v*) bromophenol blue) and were subsequently denatured at 100 °C for 10 min. Individual samples (20 μg proteins) were loaded onto 10% Tris-Acrylamide gels and electrotransferred onto polyvinylidene fluoride (PVDF) membranes (Millipore, Bedford, MA, USA). After blocking with 5% skimmed milk in TBST for 2 h at room temperature, membranes were incubated at 4 °C overnight with specific primary antibodies: PGC1-α (1:1000, CST, Beverly, MA, USA), β-actin (1:1000, CST, Beverly, MA, USA). Subsequent to washing, membranes were incubated with horseradish peroxidase-conjugated (HRP) secondary antibodies for 2 h. Signals were developed by ECL reagent (Millipore, Bedford, MA, USA) and captured by FluorChem M (ProteinSimple, San Jose, CA, USA).

### 2.4. RNA Isolation and Quantitative PCR

Total RNA was isolated from the collected cells using RNeasy Mini-kit (Qiagen, Hilden, Germany) and reverse transcribed into cDNA that prepared by PrimeScript™ RT reagent Kit (TaKaRa, Tokyo, Japan) as the manufacturer’s directions. Relative RNA expression was normalized to β-actin and miR-93-5p data was normalized to endogenous U6 small RNA using the 2-^ΔΔCt^ method. qPCR was conducted as follows: 95 °C for 10 min; and 40 cycles of 95 °C for 15 s and 60 °C for 1 min. qPCR was performed on the ABI ViiA™ 7 Real-Time PCR system (Applied Biosystems, Life Technologies, Foster City, CA, USA) using SYBER-Green qPCR Supermix (Roche Diagnostics, Basel, Switzerland). Bulge-loop™ miR-93-5p and U6 qPCR Primer Sets were purchased from Ribobio (RiboBio Co. Ltd., Guangzhou, China). Gene primer sequences were shown in Table 1.

### 2.5. Plasmids and Luciferase Reporter Assay

Wild type or mutant human *PPARGC1A* 3′UTR fragments (bases 5166-6573, NM_001330751) were synthesized by Sangon Biotech (Shanghai, China), respectively. These fragments were inserted into the multiple cloning sites of psi-CHECK-2 (Promega, Madison, WI, USA). For the luciferase reporter assay, 293T cells were co-transfected with the reporter vectors and miRNA mimics (miR-93-5p mimic or negative control (NC) mimic), or antagomirs against miRNA (anti-miR-93-5p or anti-miRNA NC). After 24 h, cells were collected and measured by using Dual-Luciferase Reporter Assay System (Promega, Madison, WI, USA) according to the kit instructions.

### 2.6. Cell Proliferation Assay and Cell Cycle Assay

Cell proliferation was measured by methylthiazolyldiphenyl-tetrazolium bromide (MTT; Sigma-Aldrich, St. Louis, MO, USA) following the manufacturer’s instructions. Twenty-four hours after transfection, cells were seeded into 96-well plates at a density of 2 × 10^3^ cells/well and incubated with 5% CO_2_ at 37 °C. At the indicated time points, cells were incubated with 20 μL of MTT solution at a final concentration of 0.5 mg/mL MTT for 4 h. After removing the supernatant, dimethyl sulfoxide (150 μL) was added to solubilize the formazan salt. After 10 min, the optical absorbance was measured at 570 nm by a plate reader (BioTek Instruments, Inc., Winooski, VT, USA). For cell cycle assays, MHCC97H or Huh7 cells were transfected with indicated oligonucleotides. At 24 h after transfection, the cells were fixed with 70% ethanol. Ethanol-fixed cells were centrifuged at 1000 rpm for 5 min, washed twice with PBS, and then incubated with 0.5 mL PBS containing 10 μg/mL RNase A and 50 μg/mL propidium iodide (PI, Sigma-Aldrich, St. Louis, MO, USA) for 30 min in the dark at 4 °C. The cell cycle distribution was analyzed using BD LSRFortessa (BD Biosciences, San Jose, CA, USA). The data was analyzed using ModFit LT3.0 software (Verity Software House, Topsham, ME, USA).

### 2.7. Statistical Analysis

The band intensity was quantified using GeneTool 4.01 software (Syngene Inc., Frederick, MD, USA). Statistical analysis was performed using the two-tailed Student’s *t*-test, and independent Student’s *t*-test was used for comparisons of two groups. The significances of the differences between the control and each experimental group was evaluated by one-way analysis of variance and the Dunnett’s post hoc test. Data was expressed as the mean ± standard deviation. *p* < 0.05 was considered to indicate a statistically significant difference.

## 3. Results

### 3.1. Extraction of microRNA-mRNA Regulatory Network

**Figure 1:**
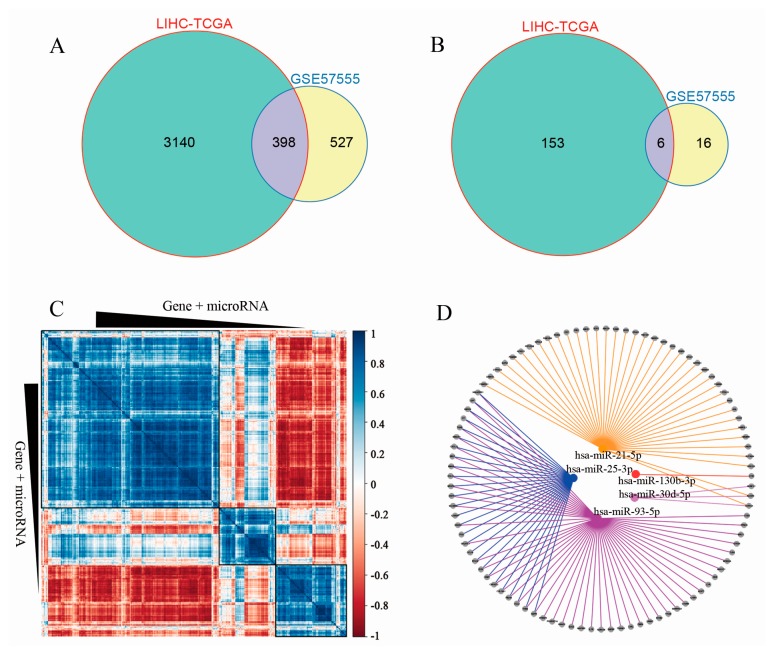
Integrated microRNA–mRNA correlation-network analysis. Venn diagram showed numbers of differentially expressed mRNAs (**A**) and miRNAs (**B**) in the different or overlapping groups; (**C**) Identifying overlapped DEGs and DEMs correlation-network in LIHC-TCGA; (**D**) Core DEGs and DEMs correlation-network based on filtering criteria (correlation coefficient r < −0.3, *p*-value < 0.05). DEGs, differentially expressed genes; DEMs, differentially expressed microRNAs; LIHC-TCGA, Liver Hepatocellular Carcinoma-The Cancer Genome Atlas.

We used GSE57555 from the NCBI GEO database and LIHC data from TCGA to construct the microRNA–mRNA regulatory network. First, we identified the DEGs and DEMs between the disease group and healthy group in GSE57555 and LIHC-TCGA, respectively. A total of 398 shared DEGs and six shared DEMs were obtained and showed by using a Venn diagram (Figure 1A,B). To extract the network, we obtained the expression values of DEGs and DEMs of LIHC-TCGA to calculate correlation values by using the correlation analysis based on Pearson’s correlation coefficient. To scale down the whole network, five DEMs and their matched mRNA targets were inversely correlated with the threshold-setting (Figure 1C,D). All of the data can be found in the supplementary data (Table S1).

### 3.2. Hub Gene PPARGC1A Was Associated with LIHC Patient Prognosis

**Figure 2:**
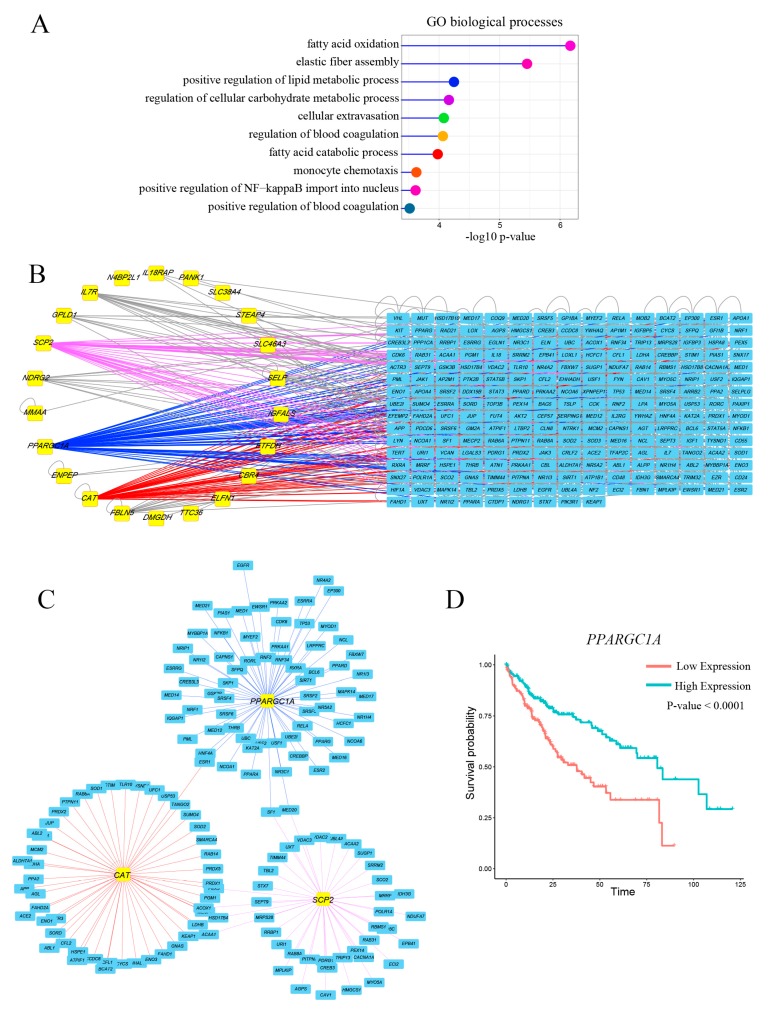
Hub gene *PPARGC1A* was associated with LIHC patient prognosis. (**A**) GO enrichment for the five DEMs matched genes. *y*-axis represents GO terms and *x*-axis represents fold enrichment (with −log10 *p*-value); (**B**) The interaction network of 22 DEGs which have significant effects on patient survival. Yellow boxes: DEGs; blue boxes: reported gene in human; (**C**) Subnetwork of three hub nodes including gene *PPARGC1A*, *CAT*, and *SCP2*; (**D**) Gene *PPARGC1A* was able to significantly distinguish the overall survival of LIHC patients. PPARGC1A: Peroxisome proliferator-activated receptor gamma coactivator-1 alpha; GO: Gene Ontology; CAT: Catalase; SCP2: Sterol carrier protein 2.

GO analysis was used to classify the related functions of five DEMs matched genes, and results showed that these genes were mainly associated with lipid metabolic process, which was consistent with the findings of previous studies (Figure 2A). In order to find the key genes, we examined the association between these genes and the survival of LIHC patients. Twenty-two genes were identified to have significant effects on patient survival (Figure S1). Subsequently, we constructed 22 genes interaction network using the BisoGenet and filtered the hub genes (Figure 2B). Generally speaking, hubs were defined as the top 15% of the nodes by degree [36]. In our study, we obtained three hub nodes, including gene *PPARGC1A*, catalase (CAT) and sterol carrier protein 2 (*SCP2*), which might play key roles in cellular processes (Figure 2C). Considering the association with survival and lipid metabolism, we eventually decided to study the correlation between gene *PPARGC1A* and miR-93-5p.

### 3.3. MiR-93-5p is Up-Regulated, PPARGC1A Is Down-Regulated in Hepatoma Cells

**Figure 3:**
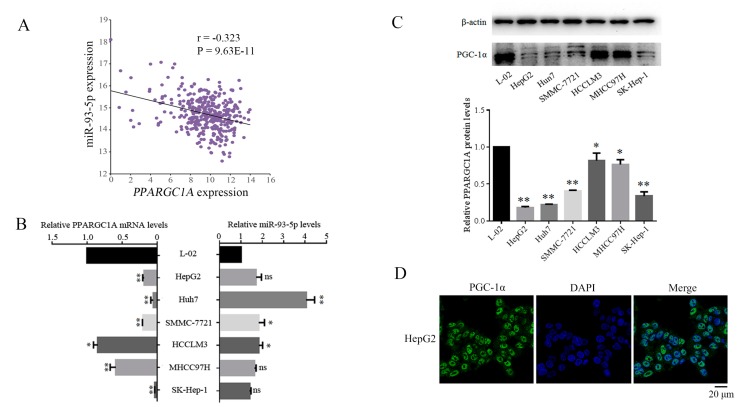
MiR-93-5p negatively inversely correlated with *PPARGC1A* expression in hepatoma cells. (**A**) A linear regression analysis was used to analyze the correlation of *PPARGC1A* and miR-93-5p expression in LIHC-TCGA; (**B**) The expression level of *PPARGC1A* mRNA and miR-93-5p were detected in hepatic cell L-02 and six hepatoma cells by qPCR; (**C**) The expression levels of PPARGC1A protein were detected in hepatic cell L-02 and six hepatoma cells by western blot; (**D**) The immunofluorescence pictures of PPARGC1A in HepG2 cell line were shown. * *p* < 0.05, ** *p* < 0.01. MiR: microRNA; qPCR: Quantitative-PCR.

The expression of miR-93-5p was inversely correlated with *PPARGC1A* mRNA expression levels in the LIHC data of TCGA (*r* = −0.323; *p* < 0.001) (Figure 3A). Next, we analyzed the expression levels of *PPARGC1A* and miR-93-5p in six hepatoma cell lines and human hepatic cell L-02 using quantitative Real-Time PCR (qRT-PCR) and western blot (Figure 3B,C). All hepatoma cell lines showed lower expression of *PPARGC1A* than L-02 cells, while there was an increase in the expression of miR-93-5p in hepatoma cell lines compared with L-02 cells, especially in Huh7 cells. In general, experimental results above suggested that miR-93-5p is up-regulated whereas *PPARGC1A* is down-regulated in the hepatoma development. In addition, we obtained the localization of PPARGC1A derived from the Human Protein Atlas database. As shown in the Figure 3D, PPARGC1A was mainly distributed in the nuclear fraction [35].

### 3.4. MiR-93-5p Suppressed PPARGC1A Expression by Directly Binding to Its 3′UTR

**Figure 4:**
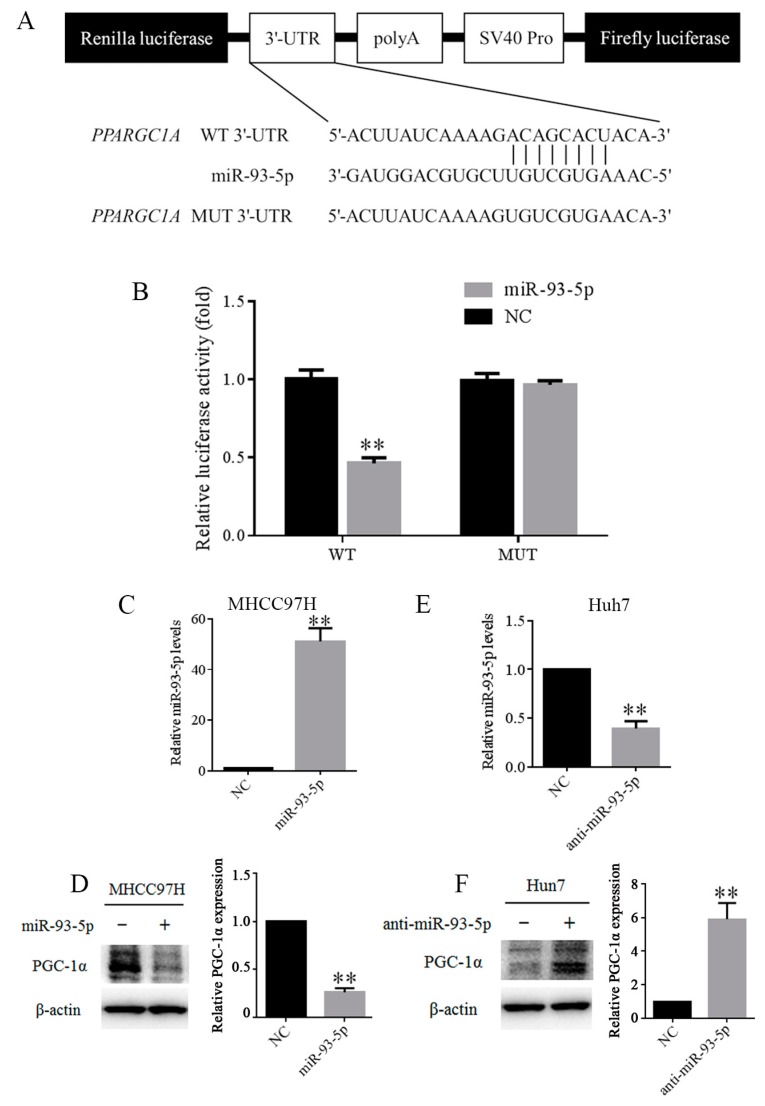
MiR-93-5p suppressed *PPARGC1A* expression by directly binding to its 3′UTR. (**A**) Schematic representation of pairing of miR-93-5p with *PPARGC1A* wild-type (WT) and mutant (MUT) 3′UTR luciferase reporter plasmids; (**B**) Relative luciferase activity in 293T cells co-transfected with WT or MUT 3′UTR *PPARGC1A* reporter plasmids and miR-93-5p or NC; (**C**, **D**) MHCC97H cells were transfected with miR-93-5p mimic and NC mimic for 48 h. MiR-93-5p levels were detected by qPCR and PPARGC1A protein levels were detected by western blot; (**E**, **F**) Huh7 cells were transfected with anti-miR-93-5p mimic and NC mimic for 48 h. MiR-93-5p levels were detected by qPCR and PPARGC1A protein levels were detected by western blot. * *p* < 0.05, ** *p* < 0.01. UTR: Untranslated region; NC: Negative control.

The 3′UTR of *PPARGC1A* mRNA contains binding sites for miR-93-5p (Figure 4A). We performed a firefly luciferase reporter assay to confirm whether miR-93-5p directly suppresses *PPARGC1A*. The 293T cells were co-transfected with the wild-type (WT) or mutant-type (MUT) 3′UTR of *PPARGC1A* luciferase plasmid, and miR-93-5p mimic or miR-NC. As shown in Figure 4B, luciferase activity of *PPARGC1A* with WT 3′UTR was significantly decreased after transfecting with miR-93-5p mimic, but not in *PPARGC1A* mRNA with the MUT 3′UTR. Indeed, western blotting analysis showed that overexpression of miR-93-5p significantly decreased the level of PPARGC1A in MHCC97H cells, while miR-93-5p inhibitor increased *PPARGC1A* expression in Huh7 cells (Figure 4C–F). These results suggested that *PPARGC1A* mRNA 3′UTR can directly be targeted by miR-93-5p.

### 3.5. MiR-93-5p Promotes Hepatoma Cells Proliferation

**Figure 5:**
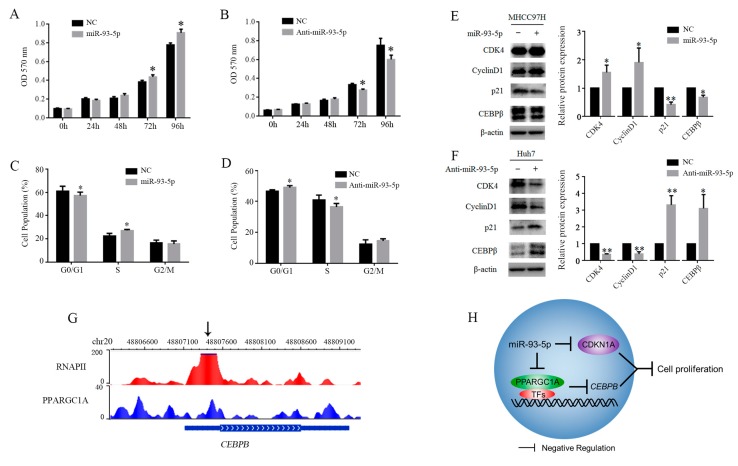
MiR-93-5p promotes hepatoma cells proliferation. (**A**,**B**) MTT assays were preformed to show the effect of miR-93-5p on hepatoma cells; (**C**,**D**) Flow cytometry assays were preformed to analyze hepatoma cells cell-cycle distribution; (**E**,**F**) Western blot analysis of protein levels of CDK4, CyclinD1, p21, and CEBPβ upon treatment with miR-93-5p or anti-miR-93-5p for 48 h; (**G**) Binding sites of *PPARGC1A* and RNAPII at the promoter regions of CEBPB gene. *x*-axis represents chromosomal positions; (**H**) Schematic representation of regulatory pathway from miR-93-5p to cells proliferation. MTT: methylthiazolyldiphenyl-tetrazolium bromide; CDK4: cyclin dependent kinase 4; CEBPB: CCAAT/Enhancer binding protein beta; RNAPII: RNA polymerase II.

We performed the cells proliferation assay and cell cycle assay to validate the biological function of miR-93-5p in the development and progression of hepatoma. The results of the MTT assay indicated that the overexpression of miR-93-5p significantly increased the proliferation of MHCC97H cells, while decreasing the miR-93-5p expression significantly inhibited the proliferation of Huh7 cells (Figure 5A,B). Moreover, the above results were also supported by cell-cycle distribution. As shown in Figure 5D, decreasing the expression of miR-93-5p led to G_1_-S arrest with the following phenotypes: the proportion of cells in the G0/G1 phase in Huh7 cells was increased compared with NC cells, and the proportion of cells in the S phase was decreased. While the overexpression of miR-93-5p in MHCC97H cells presented the opposite results (Figure 5C). Furthermore, we examined the protein expressions of cell cycle regulatory molecules to clarify the mechanisms of G_1_-S arrest. As shown in Figure 5E, the overexpression of miR-93-5p enhanced the expressions of cyclin dependent kinase 4 (CDK4) and cyclin D1, and reduced the expression of CDK inhibitor p21, while decreasing the expression of miR-93-5p presented the opposite results (Figure 5F). Moreover, we observed binding of *PPARGC1A* and RNA polymerase II (RNAPII) at the promoter regions of *CEBPB* gene by mining the public chromatin Immunoprecipitation sequencing (ChIP-seq) data of *PPARGC1A* and *RNAPII* (Figure 5G). Also, we identified that the overexpression of miR-93-5p decreased CEBPB, whereas its knockdown increased CEBPB (Figure 5E,F). Previous studies have proved that miR-93-5p inhibits *CDKN1A* genes to control HCC cell growth. Collectively, these results revealed that miR-93-5p inhibited *PPARGC1A* and *CDKN1A* genes, thereby decreasing the expressions of transcription factors CEBPB and promoting hepatoma cells proliferation (Figure 5H).

## 4. Discussion

Recently, the dysregulation of miR-93-5p has been found in various types of tumors [37,38]. Meanwhile, both miR-93-5p and *PPARGC1A* regulated similarly cellular metabolism processes. In this study, we aimed to explore the biological function of miR-93-5p and its regulation mechanism for *PPARGC1A*. Therefore, we found that miR-93-5p was up-regulated in human hepatoma cells and its overexpression promoted the proliferation of hepatoma cells. Our findings were consistent with those from previous studies. At the same time, we demonstrated that there was an inverse correlation between miR-93-5p and *PPARGC1A* expression. Furthermore, mechanism analysis suggested that miR-93-5 directly targeted *PPARGC1A* mRNA to result in hepatoma cell proliferation.

PPARGC1A is a very important transcriptional coactivator that docks to numerous TFs to regulate the expression of target genes. In addition, PPARGC1A is frequently present in a protein complex containing RNAPII [12]. As well known, PPARGC1A participates in many metabolic processes and has been implicated in several human diseases. For example, PPARGC1A activates the transcription factor peroxisome proliferator-activated receptor-α (PPARα) to enhance fatty acid metabolism [11]. Also, PPARGC1A binds transcription factor hepatocyte nuclear factor 4 alpha (HNF-4α) and glucocorticoid receptor (GR) to stimulate gluconeogenesis [10,12]. Moreover, Charos et al. utilized ChIP-seq to obtain *PPARGC1A* binding sites across the genome in hepatoma cells HepG2 [10]. Conserved motif analysis [39,40,41] showed that the majority of *PPARGC1A* binding sites are located in multiple regulatory factor binding regions including RNAPII. Additionally, these regions are frequently located at the promoter of target genes, such as genes CEBPB [42]. Indeed, we revealed that the overexpression of miR-93-5p inhibited *PPARGC1A* expression and then decreased the expression of CEBPB. Finally, hepatoma cells proliferation was enhanced. Li et al. found that silence of CEBPB promoted proliferation and inhibited the apoptosis of hepatoma cells SMMC-7721, which further supports our experimental results [43].

Previous studies have shown that miR-93-5p acts as an onco-microRNA in many types of cancer. In nasopharyngeal cancer, miR-93-5p mediated transforming growth factor-β receptor II (TGFβR2) downregulation and was associated with cancer aggressiveness [44]. In lung cancer, the overexpression of miR-93-5p inhibited disabled-2 (DAB2) to promote cell growth [45]. In gliomas, miR-93-5p mediated phosphoinositide 3-kinase (PI3K)/Akt signaling activation by directly suppresses PI3K/Tensin homology protein (PTEN), pleckstrin homology domain, and leucine-rich repeat protein phosphatase 2 (PHLPP2) and forkhead box o3 (FOXO3) expression that results in the promotion of cell proliferation [46]. In human liver cancer, miR-93-5p directly promoted cells proliferation by targeting PTEN and cyclin-dependent kinase inhibitor p21 (CDKN1A) [32]. Moreover, Thurnherr et al. [29] found that HCC-specific miRNAs (miR-21-5p, miR-93-5p, and miR-221-3p) are closely associated with cellular metabolism process, especially fatty acid metabolism. All of these results suggested that the target genes and signaling pathways of miR-93-5p are related to the specific tissue. 

## 5. Conclusions

In summary, our study reveals a novel relationship of miR-93-5p down-regulating *PPARGC1A* gene expression in HCC cells. Based on their known biological function, it is worth to further explore their correlation, molecular mechanism, and therapeutic values deeply. Taken together, we propose that the miR-93-5p–*PPARGC1A* pair regulates hepatoma progression. Both of them have clinical significance and should be considered as promising targets for hepatoma treatment in the future.

## Figures and Tables

**Table 1:** Primers sequences used for qPCR in this study.

Gene	Primer Sequence 5′ to 3′	
	Forward	Reverse
*PPARGC1A*	TGAACTGAGGGACAGTGATTTC	CCCAAGGGTAGCTCAGTTTATC
β-actin	CGTGCGTGACATTAAGGAGAAG	GGAAGGAAGGCTGGAAGAGTG

## Supplementary Materials

The following are available online at http://www.mdpi.com/2073-4425/9/1/51/s1. Table S1. DEGs and DEMs between the disease group and healthy group in GSE57555 and LIHC-TCGA. Figure S1. The association between twenty-two hub genes and the overall survival of LIHC patients.

## References

[1] Venook A.P., Papandreou C., Furuse J., de Guevara L.L. (2010). The incidence and epidemiology of hepatocellular carcinoma: A global and regional perspective. Oncologist.

[2] Stepien M., Fedirko V., Duarte-Salles T., Ferrari P., Freisling H., Trepo E., Trichopoulou A., Bamia C., Weiderpass E., Olsen A. (2016). Prospective association of liver function biomarkers with development of hepatobiliary cancers. Cancer Epidemiol..

[3] Simoneau E., Hassanain M., Madkhali A., Salman A., Nudo C.G., Chaudhury P., Metrakos P. (2014). (18)F-Fluorodeoxyglucose positron-emission tomography could have a prognostic role in patients with advanced hepatocellular carcinoma. Curr. Oncol..

[4] Eatrides J., Wang E., Kothari N., Kim R. (2017). Role of Systemic Therapy and Future Directions for Hepatocellular Carcinoma. Cancer Control.

[5] Zamora-Valdes D., Taner T., Nagorney D.M. (2017). Surgical Treatment of Hepatocellular Carcinoma. Cancer Control.

[6] Dowman J.K., Hopkins L.J., Reynolds G.M., Nikolaou N., Armstrong M.J., Shaw J.C., Houlihan D.D., Lalor P.F., Tomlinson J.W., Hübscher S.G. (2014). Development of hepatocellular carcinoma in a murine model of nonalcoholic steatohepatitis induced by use of a high-fat/fructose diet and sedentary lifestyle. Am. J. Pathol..

[7] Karagozian R., Derdak Z., Baffy G. (2014). Obesity-associated mechanisms of hepatocarcinogenesis. Metabolism.

[8] Lu J., Tan M., Cai Q. (2015). The Warburg effect in tumor progression: Mitochondrial oxidative metabolism as an anti-metastasis mechanism. Cancer Lett..

[9] Fu M., Shi W., Li Z., Liu H. (2016). Activation of mPTP-dependent mitochondrial apoptosis pathway by a novel pan HDAC inhibitor resminostat in hepatocellular carcinoma cells. Biochem. Biophys. Res. Commun..

[10] Charos A.E., Reed B.D., Raha D., Szekely A.M., Weissman S.M., Snyder M. (2012). A highly integrated and complex PPARGC1A transcription factor binding network in HepG2 cells. Genome Res..

[11] Kamimura N., Ichimiya H., Iuchi K., Ohta S. (2016). Molecular hydrogen stimulates the gene expression of transcriptional coactivator PGC-1alpha to enhance fatty acid metabolism. NPJ Aging Mech. Dis..

[12] Puigserver P., Spiegelman B.M. (2003). Peroxisome proliferator-activated receptor-gamma coactivator 1 alpha (PGC-1 alpha): Transcriptional coactivator and metabolic regulator. Endocr. Rev..

[13] Liu R., Zhang H., Zhang Y., Li S., Wang X., Wang X., Wang C., Liu B., Zen K., Zhang C.Y. (2017). Peroxisome proliferator-activated receptor gamma coactivator-1 alpha acts as a tumor suppressor in hepatocellular carcinoma. Tumour Biol..

[14] Guo L., Liang T., Yu J., Zou Q. (2016). A Comprehensive Analysis of miRNA/isomiR Expression with Gender Difference. PLoS ONE.

[15] Lin S., Cheng S., Song B., Zhong X., Lin X., Li W., Li L., Zhang Y., Zhang H., Ji Z. (2015). The Symbiodinium kawagutii genome illuminates dinoflagellate gene expression and coral symbiosis. Science.

[16] Huang Y., Zou Q., Sun X.H., Zhao L.P. (2014). Computational identification of microRNAs and their targets in perennial Ryegrass (Lolium perenne). Appl. Biochem. Biotechnol..

[17] Liu Y., Zeng X., He Z., Zou Q. (2017). Inferring microRNA-disease associations by random walk on a heterogeneous network with multiple data sources. IEEE/ACM Trans. Comput. Biol. Bioinform..

[18] Zou Q., Li J., Hong Q., Lin Z., Wu Y., Shi H., Ju Y. (2015). Prediction of MicroRNA-Disease Associations Based on Social Network Analysis Methods. BioMed Res. Int..

[19] Zou Q., Li J., Song L., Zeng X., Wang G. (2016). Similarity computation strategies in the microRNA-disease network: A survey. Brief Funct. Genom..

[20] Zeng X., Zhang X., Zou Q. (2016). Integrative approaches for predicting microRNA function and prioritizing disease-related microRNA using biological interaction networks. Brief Bioinform..

[21] Guo L., Yu J., Liang T., Zou Q. (2016). miR-isomiRExp: A web-server for the analysis of expression of miRNA at the miRNA/isomiR levels. Sci. Rep..

[22] Sun X.H., Zhao L.P., Zou Q., Wang Z.B. (2014). Identification of microRNA genes and their mRNA targets in Festuca arundinacea. Appl. Biochem. Biotechnol..

[23] Spizzo R., Nicoloso M.S., Croce C.M., Calin G.A. (2009). SnapShot: MicroRNAs in Cancer. Cell.

[24] Di Leva G., Garofalo M., Croce C.M. (2014). MicroRNAs in cancer. Annu. Rev. Pathol..

[25] Tang W., Liao Z., Zou Q. (2016). Which statistical significance test best detects oncomiRNAs in cancer tissues? An exploratory analysis. Oncotarget.

[26] Jiang L., Zhang J., Xuan P., Zou Q. (2016). BP Neural Network Could Help Improve Pre-miRNA Identification in Various Species. BioMed Res. Int..

[27] Zou Q., Mao Y., Hu L., Wu Y., Ji Z. (2014). miRClassify: An advanced web server for miRNA family classification and annotation. Comput. Biol. Med..

[28] Wang Q., Wei L., Guan X., Wu Y., Zou Q., Ji Z. (2014). Briefing in family characteristics of microRNAs and their applications in cancer research. Biochim. Biophys. Acta.

[29] Thurnherr T., Mah W.C., Lei Z., Jin Y., Rozen S.G., Lee C.G. (2016). Differentially Expressed miRNAs in Hepatocellular Carcinoma Target Genes in the Genetic Information Processing and Metabolism Pathways. Sci. Rep..

[30] Shi K.Q., Lin Z., Chen X.J., Song M., Wang Y.Q., Cai Y.J., Yang N.B., Zheng M.H., Dong J.Z., Zhang L. (2015). Hepatocellular carcinoma associated microRNA expression signature: Integrated bioinformatics analysis, experimental validation and clinical significance. Oncotarget.

[31] Liao Z., Wang X., Lin D., Zou Q. (2017). Construction and Identification of the RNAi Recombinant Lentiviral Vector Targeting Human DEPDC7 Gene. Interdiscip. Sci. Comput. Life Sci..

[32] Ohta K., Hoshino H., Wang J., Ono S., Iida Y., Hata K., Huang S.K., Colquhoun S., Hoon D.S. (2015). MicroRNA-93 activates c-Met/PI3K/Akt pathway activity in hepatocellular carcinoma by directly inhibiting PTEN and CDKN1A. Oncotarget.

[33] Plaisier C.L., O’Brien S., Bernard B., Reynolds S., Simon Z., Toledo C.M., Ding Y., Reiss D.J., Paddison P.J., Baliga N.S. (2016). Causal Mechanistic Regulatory Network for Glioblastoma Deciphered Using Systems Genetics Network Analysis. Cell Syst..

[34] Zhou X., Lowdon R.F., Li D., Lawson H.A., Madden P.A., Costello J.F., Wang T. (2013). Exploring long-range genome interactions using the WashU Epigenome Browser. Nat. Methods.

[35] Lindskog C. (2016). The Human Protein Atlas—An important resource for basic and clinical research. Expert Rev. Proteom..

[36] Guo Q., Cheng Y., Liang T., He Y., Ren C., Sun L., Zhang G. (2015). Comprehensive analysis of lncRNA-mRNA co-expression patterns identifies immune-associated lncRNA biomarkers in ovarian cancer malignant progression. Sci Rep..

[37] Li L., Zhao J., Huang S., Wang Y., Zhu L., Cao Y., Xiong J., Deng J. (2018). MiR-93-5p promotes gastric cancer-cell progression via inactivation of the Hippo signaling pathway. Gene.

[38] Xiang Y., Liao X.H., Yu C.X., Yao A., Qin H., Li J.P., Hu P., Li H., Guo W., Gu C.J., Zhang T.C. (2017). MiR-93-5p inhibits the EMT of breast cancer cells via targeting MKL-1 and STAT3. Exp. Cell Res..

[39] Liao Z., Huang Y., Yue X., Lu H., Xuan P., Ju Y. (2016). In Silico Prediction of Gamma-Aminobutyric Acid Type-A Receptors Using Novel Machine-Learning-Based SVM and GBDT Approaches. BioMed Res. Int..

[40] Liao Z., Wang X., Zeng Y., Zou Q. (2016). Identification of DEP domain-containing proteins by a machine learning method and experimental analysis of their expression in human HCC tissues. Sci. Rep..

[41] Liao Z., Wang X., Chen X., Zou Q. (2017). Prediction and Identification of Kruppel-like transcription factors by machine learning method. Comb. Chem. High Throughput Screen..

[42] Lin J., Wu P.H., Tarr P.T., Lindenberg K.S., St-Pierre J., Zhang C.Y., Mootha V.K., Jäger S., Vianna C.R., Reznick R.M. (2004). Defects in adaptive energy metabolism with CNS-linked hyperactivity in PGC-1alpha null mice. Cell.

[43] Li Y. (2015). The Effects of C/EBP-β Silence on the Proliferation, Apoptosis and Migration in Hepatocellular Carcinoma.

[44] Lyu X., Fang W., Cai L., Zheng H., Ye Y., Zhang L., Peng H., Cho W.C., Wang E., Marincola F.M. (2014). TGFbetaR2 is a major target of miR-93 in nasopharyngeal carcinoma aggressiveness. Mol. Cancer.

[45] Du L., Zhao Z., Ma X., Hsiao T.H., Chen Y., Young E., Suraokar M., Wistuba I., Minna J.D., Pertsemlidis A. (2014). miR-93-directed downregulation of DAB2 defines a novel oncogenic pathway in lung cancer. Oncogene.

[46] Jiang L., Wang C., Lei F., Zhang L., Zhang X., Liu A., Wu G., Zhu J., Song L. (2015). miR-93 promotes cell proliferation in gliomas through activation of PI3K/Akt signaling pathway. Oncotarget.

