# Supplementary figures and images for: MiR-93-5p Promotes Cell Proliferation through Down-Regulating PPARGC1A in Hepatocellular Carcinoma Cells by Bioinformatics Analysis and Experimental Verification
Source: Genes (Basel). 2018 Jan 22;9(1):51. doi: 10.3390/genes9010051 (PMC5793202; doi:10.3390/genes9010051)

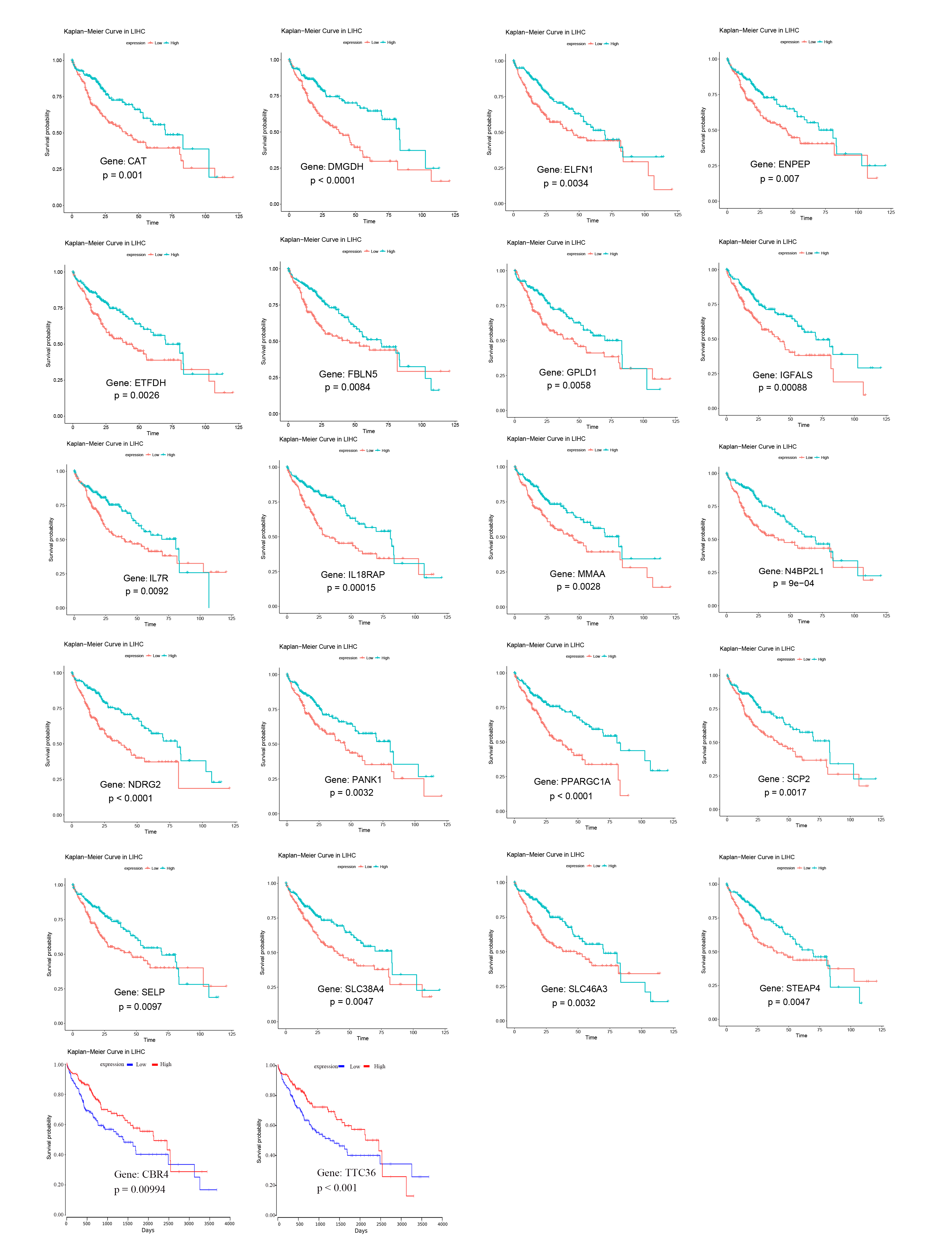

Supplement: Supplementary file 1 [file genes-09-00051-s001.zip › Supplementary Figure.tif]
